# Granulocyte-Colony Stimulating Factor (G-CSF) in Stroke Patients with Concomitant Vascular Disease—A Randomized Controlled Trial

**DOI:** 10.1371/journal.pone.0019767

**Published:** 2011-05-23

**Authors:** Agnes Floel, Tobias Warnecke, Thomas Duning, Yvonne Lating, Jan Uhlenbrock, Armin Schneider, Gerhard Vogt, Rico Laage, Winfried Koch, Stefan Knecht, Wolf-Rüdiger Schäbitz

**Affiliations:** 1 Department of Neurology, Charité-Universitätsmedizin Berlin, Berlin, Germany; 2 Center for Stroke Research Berlin, Charité-Universitätsmedizin Berlin, Berlin, Germany; 3 Cluster of Excellence NeuroCure, Charité-Universitätsmedizin Berlin, Berlin, Germany; 4 Department of Neurology, University of Münster, Münster, Germany; 5 SYGNIS Bioscience, Heidelberg, Germany; 6 HAAPACS GmbH, Schriesheim, Germany; 7 Department of Neurology, Bethel, Evangelisches Krankenhaus Bielefeld (EVKB), Bielefeld, Germany; Julius-Maximilians-Universität Würzburg, Germany

## Abstract

**Background:**

G-CSF has been shown in animal models of stroke to promote functional and structural regeneration of the central nervous system. It thus might present a therapy to promote recovery in the chronic stage after stroke.

**Methods:**

Here, we assessed the safety and tolerability of G-CSF in chronic stroke patients with concomitant vascular disease, and explored efficacy data. 41 patients were studied in a double-blind, randomized approach to either receive 10 days of G-CSF (10 µg/kg body weight/day), or placebo. Main inclusion criteria were an ischemic infarct >4 months prior to inclusion, and white matter hyperintensities on MRI. Primary endpoint was number of adverse events. We also explored changes in hand motor function for activities of daily living, motor and verbal learning, and finger tapping speed, over the course of the study.

**Results:**

Adverse events (AEs) were more frequent in the G-CSF group, but were generally graded mild or moderate and from the known side-effect spectrum of G-CSF. Leukocyte count rose after day 2 of G-CSF dosing, reached a maximum on day 8 (mean 42/nl), and returned to baseline 1 week after treatment cessation. No significant effect of treatment was detected for the primary efficacy endpoint, the test of hand motor function.

**Conclusions:**

These results demonstrate the feasibility, safety and reasonable tolerability of subcutaneous G-CSF in chronic stroke patients. This study thus provides the basis to explore the efficacy of G-CSF in improving chronic stroke-related deficits.

**Trial Registration:**

ClinicalTrials.gov NCT00298597

## Introduction

Stroke is the leading cause of adult disability in the United States and Western Europe. Two thirds of stroke survivors suffer from residual neurological deficits and have to cope with chronic motor and language dysfunctions. So far, we have very limited effective therapies in spite of intensive research efforts and numerous clinical trials [1], [2].

Basic science has improved our understanding of the mechanisms underlying recovery of function following brain injury, and started to identify strategies to facilitate these mechanisms, an important one being intensive training [3]. However, training-based interventions have so far provided only partial improvements. Limited improvement with training alone may be due to advanced age [4] and cerebral white matter lesions that lead to functional disconnection of white matter tracts in the brain [5]. Both conditions are common in the stroke population [6].

Pharmacological agents seem to be attractive tools to facilitate the beneficial effects of training-based interventions on recovery [7]. Substances only recently discussed in the context of stroke rehabilitation are neural growth factors like Granulocyte-colony-stimulating factor (G-CSF). G-CSF, a glycoprotein known to promote differentiation in the granulocytic lineage, has long been used in the clinic to counteract neutropenia and mobilize hematopoietic stem cells from the bone marrow in stem cell transplantation [8]. New data from cell culture studies and animal models of stroke have shown that G-CSF, systemically given, passes the intact blood-brain barrier and acts on neurons [8]. Importantly for the recovery phase, G-CSF not only counteracts cell death, but also exerts neuroprotective and neuroplasticity-enhancing properties, even when given at delayed time intervals [8]–[11]. Thus, GCSF may have the potential to enhance neuroplasticity leading to functional recovery even at long intervals after stroke [8], [12].

Adverse events (AEs) related to low-dose application of G-CSF in non-oncological individuals generally are only mild to moderate, including bone pain, headache, myalgias, fatigue, nausea, insomnia, and redness at the injection site. However, rare cases of potentially life-threatening conditions like splenic rupture, acute lung injury, vascular events, and exacerbation of autoimmune conditions have been reported [13]. The elevation of leukocytes, intrinsic to G-CSF treatment, may potentially lead to endothelial and blood coagulation activation causing a plugging of the microvasculature close to the infarction with an increased risk for thromboembolic events in stroke patients [13]. On the other hand, a decrease in platelet count might predispose to hemorrhagic events [13].

In the present study, our main aim was therefore to assess the safety of G-CSF in a cohort of chronic stroke patients. Patients were chosen to reflect the stroke population with regard to age, concomitant diseases like hypertension, diabetes, and generalized atherosclerosis, as well as their cerebral disease sequelae, like white matter hyperintensities (WMH). We also explored the impact of G-CSF on hand motor function for activities of daily living, motor and verbal learning, and finger tapping speed, (secondary efficacy endpoints).

## Methods

The protocol for this trial and supporting CONSORT checklist are available as supporting information; see Checklist S1 and Protocol S1.

### Ethics Statement

The study, conducted according to ICH GCP guidelines and to the principles expressed in the Declaration of Helsinki, and approved by the local ethics committee of the University of Münster and the German Federal Institute for Drugs and Medical Products, and subsequently registered on ClinicalTrials.gov (NCT00298597).

### Patient population

Fourty-one individuals (48–85 years, mean 68±8; 13 women) gave written informed consent and participated in this double-blind, placebo-controlled and randomized study. All patients received study medication for at least two days. The safety population thus consists of all 41 subjects. From two patients, no efficacy data were obtained. Thus, the full analysis set (FAS) consists of 39 subjects.

**Figure 1:**
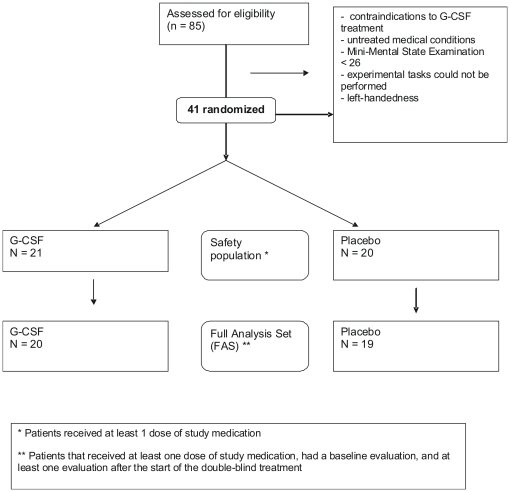
Flowchart of study. Shown is a flowchart of the trial with the number of patients in the different study populations (safety and FAS (full analysis set)).

Patients were recruited at least 4 months after having presented to the local stroke unit with an ischemic stroke, and MR-imaging of the brain had shown the presence of WMH. Severity of WMH was rated according to the validated score of Fazekas and colleagues [14] that has been widely employed in previous studies on white matter lesions in humans [15]–[17]: 0 =  no WMH, 1 =  punctate foci of WMH, 2 =  beginning confluence of foci of WMH, and 3 =  large confluent areas of WMH. Of the 85 patients that were thus eligible and agreed to participate, 41 fulfilled the additional study entry criteria: (1) No contraindications to G-CSF treatment (2) No severe and untreated medical conditions; (3) Mini-Mental State Examination >26; (4) Partial recovery from initial deficit, so experimental tasks that included fine hand motor function (Jebsen-Taylor Test) as well as a 5-finger motor sequence task could be performed; (5) Right-handedness (for further details on patients, see Table 1, Fig. 1). Demographic and stroke-specific baseline characteristics were similarly distributed between G-CSF and placebo groups.

**Table 1:** Patient Demographics.

Parameter	G-CSF	Placebo	significance
Age in years	66.4±8.9	69.9±7.2	n. s.
Gender (male/female)	14/7	14/6	n. s.
Years of education	12.2±3.1	11.7±2.3	n. s.
Fazekas-score	1.3±0.5	1.3±0.6	n. s.
Weeks since stroke	52.9±12.1	64.0±36.0	n. s.
NIHSS	1.2±1.1	1. 2±1.5	n. s.
Modified Rankin scale	1.2+0.8	1.3+1.1	n. s.
Lesion side (left/right)	10/11	9/11	n. s.
Arterial hypertension	18	16	n. s.
Diabetes mellitus	6	5	n. s.
At least one additional vascular risk factor (Hypercholesterolemia, coronary heart disease, peripheral arterial occlusive disease)	15	16	n. s.
Blood pressure (systolic/diastolic) [mmHg] at baseline	131/78±15.6/12.3	135/81±15.6/9.0	n. s.
heart rate [beats/minute] at baseline	72±14.3	72±11.7	n. s.
body temperature [°C] at baseline	35.9±0.5	36.0±0.4	n. s.

n. s.: not significant; NIHSS: National Institutes of Health Stroke Scale; mean ± SD given; significance determined by t-test, Mann-Whitney, or chi^2^ test as appropriate.

All patients were evaluated neuropsychologically in a separate session before G-CSF administration, using tests of general intellectual function, attention, verbal fluency, digit spans, and verbal and visuospatial memory. Patients were recruited into the study from June 2006 to August 2008.

### Study medication

Study medication, and randomization to either G-CSF or placebo, was provided by the pharmacy of the University of Mainz. Filgrastim (recombinant human G-CSF produced in E. coli, solubilised in a buffer containing 10 mM acetic acid, 5% (m/v) sorbitol, 0.004% Tween 80, ph adjusted to 4.0 with NAOH), or saline as placebo were filled in identical-looking glass vials to ensure blinding. Treatment was then administered by the study nurse. Results of blood analysis and potentially medically necessary actions triggered by elevated leukocyte counts were exclusively accessible to study investigators who did not take part in testing and analysis of efficacy parameters, or rating of adverse events.

### Study outline

**Figure 2:**
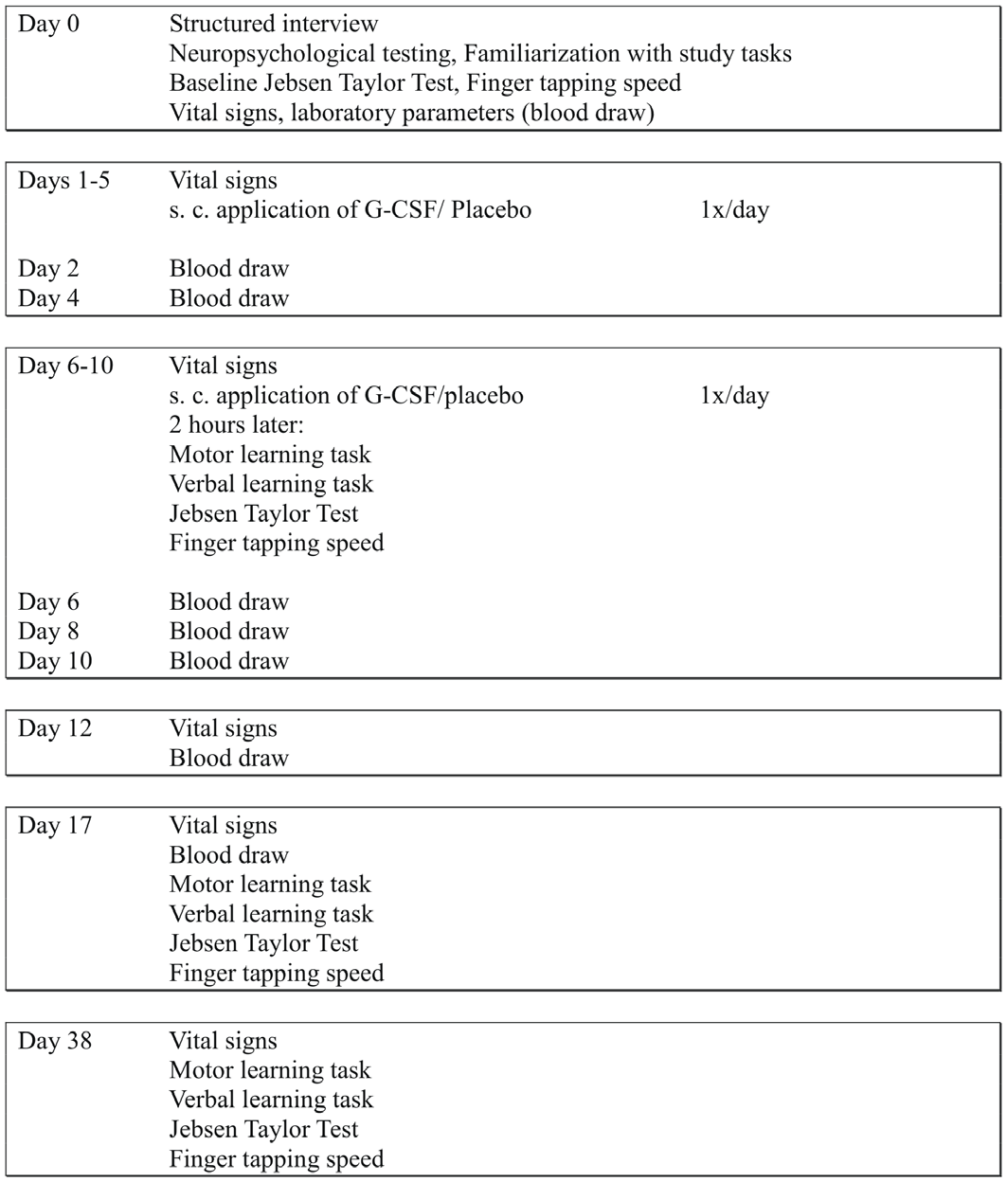
Time schedule of procedures and tests performed. Shown are the different test series performed over 38 days.

Patients underwent neuropsychological testing and familiarization in all tasks one to three days before receiving the first dose of G-CSF or placebo. Then, each participant was given either G-CSF (10 µg/kg body weight/day), or saline solution (placebo, 0.1 ml/kg body weight/day), each administered as daily s. c. injections over 10 days. This dose is in line with literature safety data for healthy human bone marrow donors [18]. Vital signs and laboratory parameters were determined repeatedly throughout the course of the study (see Fig. 2 for details). Additionally, the trial assessed the efficacy of G-CSF versus placebo on motor hand functions in hand activities of daily living, motor and verbal learning, and finger tapping speed.

### Efficacy parameters

To assess motor hand functions, the Jebsen Taylor Test (JTT) was employed [19]. This test has been widely used to assess a broad range of hand functions required for activities of daily living, has good validity and reliability, and has been extensively used in rehabilitative settings [19]–[21]. Six subtests were performed: Turning over cards, picking up small objects and placing them in a can, picking up small objects with a teaspoon and placing them in a can, stacking checkers, moving large light, and moving large heavy cans. Dependent variable was total time to complete the six subtests.

To assess motor learning, we used the serial reaction time task in a modified version with a probabilistic instead of a deterministic sequence to ensure the procedural task nature [22], [23] (15% random and 85% sequential elements in each block). Patients sat in front of a 14-inch monitor with their right hand placed on a special keypad with 5 different keys, one for each finger. Following the rules of a finite-state grammar [22], [23], one of the black squares on the screen was replaced by an asterisk, and patients had to press the corresponding keys as fast as possible. The task consisted of 2 blocks of 500 key-presses each. Dependent variable was difference in reaction times between random and sequential elements.

To assess verbal learning, a word learning model was employed that mimics vocabulary acquisition in healthy individuals, and is relevant to language reacquisition in stroke patients with aphasia [24], [25]. Patients had to indicate by button presses whether they deemed a particular coupling to be correct or incorrect. The underlying learning principle was higher statistical co-occurrences of certain couplings as compared with other pairings. Participants were trained for five days, each day about 50 minutes. Details of the training program are described elsewhere [24]. Dependent variables were percentage of correct responses and reaction times.

To assess finger tapping speed, patients performed a fast finger-tapping task [26]. Patients were instructed to press a key with the right index finger as quickly as possible for a total of 10 seconds. The task was repeated three times, with 1-minute resting intervals between trials. The keypad was connected to a laboratory computer that recorded the frequency of tapping (dependent variable).

### Statistical analysis

Sample size was derived from the AXIS I study [27], and from similar studies using experimental treatments in chronic stroke patients [28], [29]. Primary endpoint was the total number of adverse events (AEs), as a function of group (unpaired t-test). Secondary safety endpoints were number of treatment-related adverse events (AEs), discontinuation due to treatment-related AEs, laboratory parameters including leukocyte, erythrocyte, and platelet count, and vital signs (body temperature, blood pressure, heart rate). Judgment of causality was conducted by an investigator of the trial (WRS). According to the study protocol, the following points were considered: 1) Known pharmacology of G-CSF; 2) Side effects described in the product information of G-CSF; 3) Side effects described in the literature; 4) Timely relation to treatment with G-CSF. The safety population consisted of all subjects who received at least one dose of study medication (n = 41).

Additionally, secondary efficacy endpoints were explored, that is, the changes from Day 3 to mean of Day 17 and Day 38 for JTT and finger tapping speed, as well as success in verbal and motor learning during the second week of treatment, as a function of group. Here, the pre-defined primary efficacy endpoint was the JTT. The analyses were performed in the full analysis set according to guideline ICH E9 (FAS) population, which was defined as all subjects who received at least one dose of study medication, had a baseline evaluation, and at least one evaluation after the start of the treatment (n = 39). Last observations were carried forward in the endpoint analysis (LOCF). Analysis of covariance with adjustments for age, time since infarction, location, lesion size, and baseline value was performed for the efficacy variables. Significance for all tests was set at p<0.05. All data are expressed as means ± SEM, unless stated otherwise. All statistical analyses were performed using SPSS 16, JMP 8.01, or SAS 9.1.

## Results

### Subjects included in analyses

All 41 subjects originally recruited into the study took at least two doses of study medication (safety population). Two patients that received at least one dose of study medication had to be excluded, one of them due to acute disease unrelated to the study (bronchitis), the other withdrew consent without specific reason. Thus, 39 subjects remained for the efficacy analyses (see Figure 1, flowchart of patient populations).

### Safety analysis: Adverse events

All patients (n  = 41) received at least one dose of study medication. In general, the study medication was tolerated well. In three patients receiving verum the dose was halved at day 10 (2) or day 3 (1) due to leukocyte count >50/nl without clinical symptoms. In three patients medication was halted at day 9 (2) or day 8 (1) due to leukocyte count >70/nl without clinical symptoms. In two patients verum medication was stopped at day 3 or day 5 due to bronchitis and headache cum nausea, respectively. One patient received dosing every second day starting at day 4 due to development of bone pain that was manageable under this regimen.

Three patients in the G-CSF group (3/21) and one patient in the placebo group (1/20) suffered from serious AEs (SAEs), none of which was judged to be related to study medication. These SAEs were: Pons infarction, bronchitis, bone fracture, and infection with unclear source. The number of patients in each group with at least one AE was 17/21 in the G-CSF and 14/20 in the placebo group (Fisher's Exact Test, p = 0.48). The total number of AEs was 58 for verum, and 37 for placebo (Chi Square Test p = 0.055). AEs in the G-CSF group were more often related to the treatment itself: 33/58 (59%) events reported in the G-CSF group were classified as likely related to the treatment, versus 18/37 (49%) in the placebo. This effect revealed a strong trend for more related events in the G-CSF group when comparing absolute numbers (Chi Square Test p = 0.054). All treatment-related events were rated in their severity as mild or moderate, and were within the known side effect profile of G-CSF treatment, most often headache (n = 9), fatigue (n = 9), malaise (n  = 6), back pain (n = 5), limb discomfort (n = 3), and abdominal pain (n = 1). Nausea (n  = 4), and sleep disorders (n = 7) were rated as not treatment-related; other side effects occurred with a frequency of<2. Most frequent side effects in the placebo group included headache (n = 7), fatigue (n = 8), and dizziness (n = 5). For further information on adverse events, please refer to Table 2 and 3. In conclusion, a higher number of mild to moderate AEs related to treatment were observed in the G-CSF group, explained by the known side effect profile of this growth factor.

**Table 2:** All adverse events, listed by system organ class (n = 21 in the G-CSF, n = 20 in the placebo group.

Side effects	AEs		Patients with AEs	
	Placebo	G-CSF	Placebo	G-CSF
Nervous system disorders	17	14	11	10
General disorders and administration site conditions	8	15	5	10
Musculoskeletal and connective tissue disorders	3	7	2	7
Gastrointestinal disorders	1	8	1	5
Psychiatric disorders	0	7	0	5
Respiratory, thoracic and mediastinal disorders	0	4	0	4
Infections and infestations	1	2	1	2
Skin and subcutaneous tissue disorders	3	0	3	0
Vascular disorders	1	1	1	1
Eye disorders	1	0	1	0
Investigations	1	0	1	0
Surgical and medical procedures	1	0	1	0

**Table 3:** All treatment-related adverse events (probable or possible), listed by system organ class (n = 21 in the G-CSF, n = 20 in the placebo group).

Side effects	Events		Patients	
	Placebo	G-CSF	Placebo	G-CSF
**General disorders and administration site conditions**	8	15	5	10
**Nervous system disorders**	7	12	5	8
**Musculoskeletal and connective tissue disorders**	1	6	1	6
**Investigations**	1	0	1	0
**Skin and subcutaneous tissue disorders**	1	0	1	0

### Safety analysis: Vital signs and laboratory values

Analysis of blood pressure, heart rate, and body temperature revealed no significant difference between groups at baseline, at Study Days 6–10, Study Day 17, or 38.

**Figure 3:**
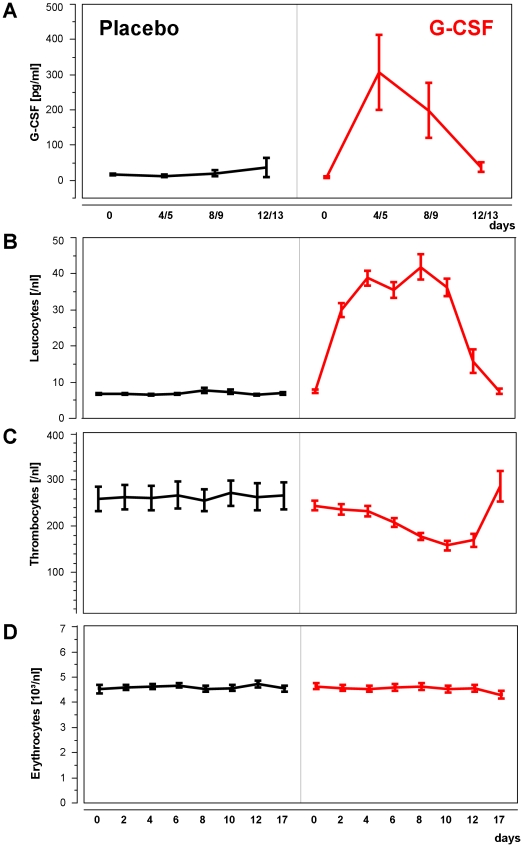
G-CSF levels and hematological parameters. **a,** serum concentrations of G-CSF measured at 4 time points throughout the study. Two days after the end of treatment (day 12/13) levels have returned to normal. Levels were measured at day 0, day 4 or 5, day 8 or 9, and day 12 or 13. **b-c,** hematological parameters. **b,** Leukocytes are already elevated at day 2, and reach their peakk at day 8. 7 days after end of medication levels have returned to normal (day 17). Error bars indicate standard errors of the mean (SEM). **c,** Thrombocyte counts decrease under G-CSF treatment. Lowest numbers are reached by day 10. Numbers normalize 7 days after end of treatment. **d,** No influence of treatment on erythrocyte numbers was detected.

We measured G-CSF values at day 0, days 4 or 5; days 8 or 9, and day 12/13. G-CSF levels in the verum group rose from 9.26±2.42 pg/ml serum at baseline to 306.07±106.81 pg/ml at day 4, and were already back to 37.83 ±13.68 pg/ml at day 12/13 (Figure 3a).

As expected, leukocyte count showed a significant increase in the verum group, from mean 7.5/nl at baseline to a mean value of 41.9/nl on day 8. Highest leukocyte count, reached in one patient on day 8, was 75.4/nl. At day 17 (7 days after last administration of G-CSF/placebo) values were back to normal (mean 7.42±0.72/nl for G-CSF vs. 6.94±0.34/nl for placebo (Figure 3b).

We observed an anticipated drop in platelet count, with a decline starting on study day 2, reaching its lowest count on study Day 10, and returning to baseline on study day 17 (Figure 3c). The difference between treatment groups became significant from day 8 to 12 (all p's<0.008). Lowest platelet count was reached at day 10 with 158.26±9.94 thrombocytes/nl.

For erythrocyte count, no major difference between treatment groups was found (Figure 3d).

### Influences on motor and cognitive measures

**Figure 4:**
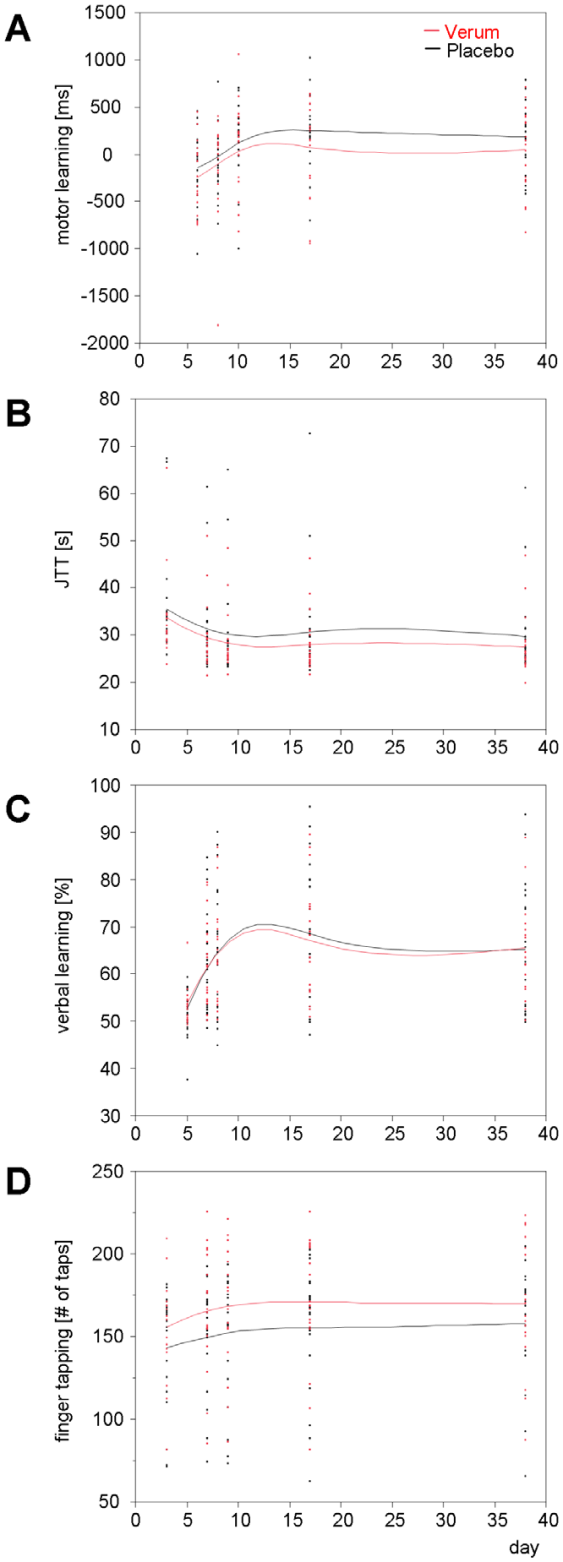
Exploratory efficacy outcome measures. **a,** motor learning task: Magnitude of learning is reflected by the difference in reaction times between random and sequential elements [ms]. **B,** Jebsen Taylor Test of hand function: Performance in this test was reflected by total time needed to complete the six subtests [s]. **c,** verbal learning task: Magnitude of learning is reflected by the [percentage] of correct responses (words learned). **D,** finger tapping speed: Performance in this test was reflected by the [number of key presses] within the time interval. Curves were fitted using a cubic spline function with a lambda of 0.05.

Patients included in the efficacy analysis were able to perform all four behavioural tasks. Behaviour of treatment groups over time in tests for JTT, motor learning, verbal learning, and finger tapping speed appeared similar (Figure 4a–d). As the pre-defined primary efficacy endpoint, difference between G-CSF and placebo in change from JTT at Day 3 to study end point (mean of Day 17 and 38) did not reveal a significant effect of treatment (6.0 s±0.9 in the G-CSF group, 5.5 s±0.8 in the placebo group). Number of errors was likewise comparable between groups (t-test, ns). In an extended exploratory statistical model including covariates age, time since infarction (statistically significant), lesion size, side of infarction, and JTT at Day 3, a similar result emerged for difference between treatment groups (p = 0.36). Our primary endpoint for exploratory efficacy analysis was therefore negative.

## Discussion

The present study shows that subcutaneous G-CSF treatment in elderly chronic stroke patients with concomitant vascular disease is safe and reasonably well tolerated. Analysis of exploratory motor and cognitive measures did not detect significant effects of treatment in the pre-specified analyses.

So far, only data on two randomized controlled trials with G-CSF application in stroke patients are available [30]. A trial in acute stroke patients (AXIS) demonstrated the safety of a 3-day course of high-dose G-CSF, administered intravenously [27]. Incidence of thromboembolic events and distribution of AEs were similar between treatment groups, and no negative influence on the development of vessel occlusion or stenosis from baseline to end of treatment was noted. Thus, the concern about harmful effect on vessel walls raised previously [31] was not substantiated. Two previous pilot studies in acute and subacute stroke patients likewise suggested safety of G-CSF treatment [30], [32].

Our study is the first randomized controlled trial that assessed the safety and feasibility of G-CSF in chronic stroke patients, over an extended administration period (10 days). Numbers of all AEs, and of likely related AEs, the primary endpoint aimed at safety, were more frequent in the G-CSF group although this difference failed formal significance. However, this is not unexpected given the known side effect spectrum of G-CSF, especially with the longer time frame of administration in comparison to the recent AXIS trial. Importantly, AEs with likely relationship to study medication were always rated as mild to moderate, and all involved well-known side effects from G-CSF. In two patients, likely related adverse events (bone pain and headache cum nausea) led to intermittent dosing or to discontinuation of study medication.

A rise in leukocytes is the main expected effect of G-CSF outside the central nervous system. Leukocytes reached an average maximum of 42/nl at day 8, and dropped back to baseline levels less than one week after end of treatment. Although no clinical data exist that ascribe harmful effects to an isolated, G-CSF-triggered rise in leukocytes, we halved dosing at leukocyte counts >50/nl, and halted dosing at values >70/nl.

Similar to the study in acute stroke (AXIS), the rise in leukocytes did not lead to an increase in thromboembolic events. It is reassuring that so far no clinical evidence suggests a G-CSF treatment-related adverse effect on the cerebral microvasculature assessed in almost hundred stroke patients [27], [30], [31]. This observation is backed by a recent animal study revealing no evidence for microvascular plugging or thrombembolic adverse events despite a significant rise of white blood cells in the peri-ischemic microcirculation [33].

Apart from the rise in leukocytes, a lowering in platelets was noted, which is a known side effect of G-CSF treatment also in other patient populations. Thrombocyte counts did rapidly recuperate to normal levels 7 days after end of G-CSF treatment. However, in future studies with chronic G-CSF treatment this parameter needs to be closely monitored, especially in patients receiving anticoagulation or platelet aggregation blockers.

In summary, the present study suggests safety and reasonable tolerability of G-CSF application over the course of 10 days, even in the vulnerable population of elderly stroke patients with concomitant diseases like hypertension, diabetes, and WMH, known to increase morbidity and mortality [34]. This finding is in line with the data from 20 years of clinical oncological experience with G-CSF that has demonstrated an excellent clinical safety profile for the substance (see [18] for review).

However, it needs to be clarified if higher elevation of leukocyte count than 50 – 70/nl can be tolerated in chronic application, and lowering of platelets needs to be closely monitored.

We have conducted a broad array of tests in view of demonstrating feasibility of such a trial approach, but also with the aim to explore possible efficacy signals. The tests explored did not show a meaningful difference between G-CSF and placebo, including the Jebsen-Taylor test (JTT), chosen as primary efficacy parameter.

Several reasons may be responsible for the failure to detect clearer signals of efficacy in our study. First, the current small study is certainly underpowered to show effects in a highly heterogeneous population as the one examined here. This is underlined by a retrospective power analysis: We had a 25% chance of detecting a 25% effect size in the main outcome parameter, change in JTT from day 3 to mean of day 17 and day 38 (population change = 6.1 points±3.6 SD), and an 8% chance to detect an effect size of 10%. A sample size of 176 is needed to detect a 25% effect size with a power of 80%. Second, we chose patients that had almost completely recovered from their initial deficits, and were therefore able to participate in the experimental tasks. However, some patients were more than a year away from their stroke, and larger effects of G-CSF may have been present if G-CSF had been given at an earlier stage [9]. Third, patients had to perform the task always with their dominant right hand, thus avoiding an interaction of intervention effect with handedness [23], [35]. Fourth, with the primary focus to assess safety of G-CSF in chronic stroke, the study included patients with different lesion sizes (lacunar, small territorial) and sides (right and left) and different time intervals from the stroke event. These particulars of the study design might have contributed to diluting a possible beneficial effect of G-CSF on learning in the verbal or the motor domain, or motor function. Future studies on efficacy should therefore include a more homogeneous patient cohort, and be conducted at an earlier state after stroke. Note also that hand motor function had its “baseline assessment” on day 3 only, and motor and verbal learning were assessed during the second week of treatment, to detect differences in learning ability under G-CSF versus placebo. A significant difference between treatments may have emerged if baseline assessment of hand motor function had been conducted at day 0, or pre-treatment learning had been compared with learning during treatment for both G-CSF and placebo condition, a hypothesis that should be explored in future studies.

Concomitant white matter lesions might also have complicated benefit assessment in our population: Efficient communication in the human brain relies on the integrity of white matter tracts [36], [37], and severity of WMH is negatively associated with rehabilitation succession in neurological disease [38], possibly due to disconnection of cerebral circuits, reduced vascular density and cerebral blood flow, and impaired synaptic plasticity (see [38] for review).

Apart from particular limitations of the study cohort and power issues, questions of dosing and timing are evident. Higher doses should be included in the next trial, as an increase of effect size seems to occur with dose in acute stroke patients (AXIS). Also, a longer time period or repeated dosing intervals may be needed to see sustained effects.

In summary, the present study is a further indication that G-CSF administration is safe and reasonably well-tolerated, even in elderly chronic stroke patients with concomitant vascular disease. It has to be kept in mind though that only a small number of patients (n = 21 in the G-CSF group) were examined. However, this safety analysis is the first step on the road to introducing a novel therapeutic approach to the clinical realm [39].

Given the long-time experience with G-CSF in other indications, and the strong rationale for G-CSF as a recovery-enhancing drug it is now the time to initiate adequately powered trials examining the potential efficacy of this drug in improving functional outcome in the subacute or chronic stage after stroke.

## Supporting Information

Checklist S1 **CONSORT Checklist.**
(DOC)

Protocol S1 **Trial Protocol.**
(DOC)
