# Supplementary material for: Granulocyte-Colony Stimulating Factor (G-CSF) in Stroke Patients with Concomitant Vascular Disease—A Randomized Controlled Trial
Source: PLoS One. 2011 May 23;6(5):e19767. doi: 10.1371/journal.pone.0019767 (PMC3100298; doi:10.1371/journal.pone.0019767)
Supplement: Protocol S1 — Trial Protocol. (DOC) [file pone.0019767.s002.doc]

**G-CSF in the treatment of stroke**

Prof. Wolf-Rüdiger Schäbitz, MD

Dr. Thomas Duning, MD

Dr. Tobias Warnecke, MD

University Hospital of Münster, Germany

**1. Aim of the study and Introduction**

- *Why is the study conducted?*
The long term goal of the study will be the pharmacological treatment of stroke sequelae by drugs that stimulate neurogenesis in the central nervous system (CNS) and have neuroprotective effects.

The immediate objective of the study will be to demonstrate that systemically applied haematopoietic growth factors increase neurogenesis and lead to a neuroprotection in stroke patients, shown as an improvement of motor functions and cognitive functions.
 *-What is known?*
Various animal experiments in recent years have shown that the adult brain is able to produce new nerve cells (neurogenesis). The old hypothesis that the formation of new nerve cells at birth is complete was proved to be obsolete [Kempermann et al. 1998].

Neurogenesis particularly takes place in the subventricular zone and dentate gyrus of the hippocampus, a brain region which is important for learning and remembering. Every day several thousands of so-called "granule -cells" in the hippocampus of mammals evolve [Roy et al. 2000, Jin et al. 2003, Nunes et al. 2003, Kempermann et al. 2004].

The neurogenesis in the adult brain is modulated by various environmental factors, such as physical activity, mental stress or learning [Nilsson et al. In 1999, Van Praag et al. 1999 Döbrössy et al. 2003, Drapeau et al. 2003 Dragansky et al. 2004]. Neurogenesis in the adult brain can also be stimulated by subcutaneous or intravenous administration of pharmacological substances. Animal studies have shown that systemic administration of various peripheral growth factors (Basic Fibroblast Growth Factor, Insulin-like growth factor-I) leads to an increased neurogenesis in the subventricular zone and dentate gyrus of adult rats [Wagner et al. 1999, Aberg et al. 2000].

Hemato-and granulopoietic growth factors, such as erythropoietin (EPO) and granulocyte - colony stimulating factor (G-CSF) do not only control the proliferation and differentiation of progenitor cells of hematopoiesis, but also have a neuroprotective and neurotrophic effect. Several animal studies have shown that there were different effects of EPO and G-CSF on neurons. These effects are mainly mediated by specific receptors (EPOR and G-CSFR). Therefore, it is postulated that the brain might have a specific, local EPO / EPOR and G-CSF/G-CSFR – system [Dame et al. 2001, Sirén et al. 2001 Schäbitz et al. 2003, Ghezzi et al. 2004, et al Jelkmenn. 2004, Maiese et al. 2005 Schäbitz et al. (Unpublished results)]. Since G-CSF can cross the blood-brain barrier, a direct effect of systemically administered G-CSF to the cerebral G-CSF receptor is possible, [Schäbitz et al. (Unpublished results)]. Experimental studies have demonstrated that treatment with G-CSF in mice or rats with brain ischemia can improve motor function [Schäbitz et al. 2003, Shyu et al. 2004, Gibson et al. 2005 Schäbitz et al. (Unpublished results)]. Even mice with an autoimmune encephalomyelitis have benefited from a treatment with growth factors [Zhang et al. 2005].

The expression of growth factors in the cortex and in the hippocampus decreases with age [Chung et al. 2004]. In animal experiments we could demonstrate that G-CSF stimulates neurogenesis in the dentate gyrus of adult rats, which points to a promoting effect of neurogenesis in the adult brain [Wang et al. 2004].
The influence of neurogenesis in the adult hippocampus to memory functions is not yet clear. However, it was recently demonstrated that newly generated neurons in the adult mammalian brain could mature to functioning neurons [Van Praag et al. 2002].

Stroke is the leading cause of adult disability in the United States and Western Europe.Two thirds of stroke survivors suffer from residual neurological deficits and have to cope with chronic motor and language dysfunctions. After completing standard motor rehabilitation, about 50–60% of stroke patients still experience some degree of motor impairment. Motor deficits impair activities of daily living, such as dressing, engaging in self-care, and communicating. Persisting language deficits (aphasia) after stroke, affecting approximately 18% of stroke patients, contribute significantly to permanent disability and emotional suffering in patients. Because of their communicative disability, the majority of patients with chronic aphasia are unable to maintain their previous jobs and suffer from a reduction of social contacts. Taken together, the motor and language sequelae of stroke greatly impact individual patients and their families, and put a large burden onto public health systems. So far, we have very limited effective therapies in spite of intensive research efforts and numerous clinical trials.

*- Prior studies?*Several animal studies exist that have shown the neuroprotective and neurotrophic effects of G-CSF [Schäbitz et al. 2003]. In these studies systemically applicated G-CSF - similar to EPO – prevented the programmed cell death (apoptosis counteracts) of nerve cells, and thus demonstrated a neuroprotective effect. Furthermore, it was shown that the expression of G-CSF/G-CSFR increased after G-CSF application in ischemic brain tissue (especially in the penumbra). Treatment with G-CSF has resulted in a reduction of infarct volume and improved clinical outcome (recovery of sensory-motor functions). Interestingly, in the preliminary animal studies have also demonstrated that the administration of G-CSF do not only increase neurogenesis in the dentate gyrus of experimental animals suffering from a stroke, but also in all healthy animals.

*-What should be investigated?*
This study is designed to assess the safety of G-CSF in a cohort of chronic stroke patients. We will also explore the impact of G-CSF on motor and verbal learning, hand motor function for activities of daily living, and finger tapping speed (secondary efficacy endpoints).

**2. Experimental design and methods**

**Study outline**

Patients will undergo neuropsychological testing, as well as familiarization in all tasks, one to three days before receiving the first dose of G-CSF or placebo. Then, each participant will be given either G-CSF (10 µg/kg body weight/day), or saline solution (placebo, 0.1 ml/kg body weight/day), each administered as daily s. c. injections over 10 days. This dose is in line with literature safety data for healthy human bone marrow donors. Vital signs and laboratory parameters will be determined repeatedly throughout the course of the study (see Figure on study design below). Additionally, we will assess the efficacy of G-CSF versus placebo on motor and verbal learning, and motor hand functions in hand activities of daily living.

**Efficacy parameters**

To assess motor learning, we will use the serial reaction time task in a modified version with a probabilistic instead of a deterministic sequence to ensure the procedural task nature (15% random and 85% sequential elements in each block). Patients will sit in front of a 14-inch monitor with their right hand placed on a special keypad with 5 different keys, one for each finger. Following the rules of a finite-state grammar, one of the black squares on the screen will be replaxed by an asterisk, and patients have to press the corresponding keys as fast as possible. The task consists of 2 blocks of 500 key-presses each. Dependent variable will be difference in reaction times between random and sequential elements.

To assess verbal learning, a word learning model will be employed that mimics vocabulary acquisition in healthy individuals, and is relevant to language reacquisition in stroke patients with aphasia. Patients have to indicate by button presses whether they deem a particular coupling to be correct or incorrect. The underlying learning principle will be higher statistical co-occurrences of certain couplings as compared with other pairings. Participants will be trained for five days, each day about 50 minutes. Details of the training program are described elsewhere. Dependent variables are percentage of correct responses and reaction times.

To assess motor hand functions, the Jebsen Taylor Test (JTT) will be employed. This test has been widely used to assess a broad range of hand functions required for activities of daily living, has good validity and reliability, and has been extensively used in rehabilitative settings. Six subtests will be performed: Turning over cards, picking up small objects and placing them in a can, picking up small objects with a teaspoon and placing them in a can, stacking checkers, moving large light, and moving large heavy cans. Dependent variable will be total time to complete the six subtests.

To assess finger tapping speed, patients will perform a fast finger-tapping task in both sessions. Patients will be instructed to press a key with the right index finger as quickly as possible for a total of 10 seconds. The task will be repeated three times, with 1-minute resting intervals between trials. The keypad will be connected to a laboratory computer that records the frequency of tapping (dependent variable).

The study design is shown below.


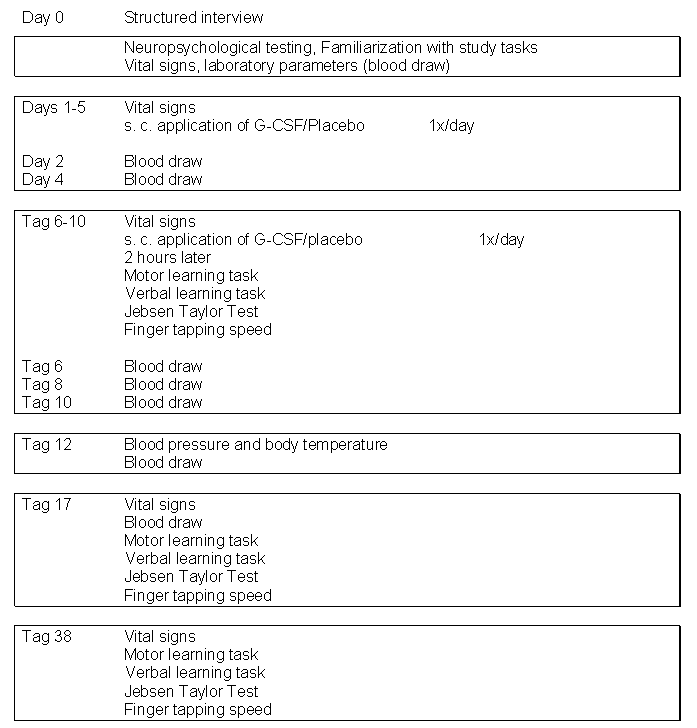


*- What is the design of the study? Is it*

*- A pharmacological, clinical pharmacology, clinical or other biomedical study?*Clinical-pharmacological (administration of growth factors) and biomedical (measurement of safety data, motor and cognitive function) study.

- *An open or single-or double-blind study?*
It is a placebo-controlled, double-blind study.

*- Randomized study?*The allocation of subjects to the different test substances will be randomized.

*- Diagnostic, therapeutic, compatibility or exclusive scientific study?*It is a scientific and therapeutic study.

*- By whom will the participants be medically supervised before, during and after study? (Agreement with the GP, control of other medications?)*The supervision of the study participants is made by the investigator, Dr. Tobias Warnecke, medical resident at the Department of Neurology, University Hospital of Münster (UKM).

*- Duration of the study*The study lasts one to three years.

*- Will interim results be analyzed to detect a trend? What criteria and which consequences are drawn for the participants?*Because of the double-blind nature of the study no interim results can be analyzed.

- *What kind of documentation is provided?*

Clinical data will be stored as data files. For each subject a test code will be assigned. The assignment list of drugs and codes is maintained by the Pharmacy of the University Hospital of Münster.

*- Is the involvement of a statistician provided and what statistical methods will be used?*Primary endpoint will be the total number of adverse events, as a function of group (unpaired t-test). Secondary safety endpoints will be number of treatment-related adverse events, discontinuation due to treatment-related AEs, laboratory parameters including leukocyte, erythrocyte, and platelet count, and vital signs (body temperature, blood pressure, heart rate).

- *Will the medical confidentiality and privacy provisions be respected?*
The medical confidentiality and privacy provisions are respected.

*- Will the trial participants get a fee (expenses paid, etc.)? How much?*
No.

*- Will the participating investigator get a fee? How much?*
No.

- *Number of participants (in case of comparative studies please name the number of patients in each groups sharing)*A total of up to 40 subjects should be included that complete the study (safety, efficacy data), 20 in each of the two groups.

- *Age and Gender of participants (please indicate the age of the participant and the upper and lower limits of the exclusion criteria)*Adult patients (aged 18 or older, no upper age limit), men and women.

Patients at least 4 months after ischemic stroke, who suffer from cerebral vascular disease sequelae, like white matter hyperintensities, will be included.

*- Status: Are the participants
 a) healthy persons?* No.

 *b) pregnant or breastfeeding women?* No.
 *c) relevant sufferers? (Please specify the disease and the state)*
 Patients after ischemic stroke, with cerebral vascular disease sequelae, like white matter hyperintensities

 *d) Inpatient or outpatient?* Outpatients

 *e) Persons suffering from other diseases? (In particular mental illness, and are there doubts about the business or legally competence?)* No

*- What are other inclusion criteria (for example: allowed concomitant medications)?*Partial recovery from initial deficit so experimental tasks could be performed; and Right-handedness.

- *What other exclusion criteria (such as advanced kidney or liver failure, etc. prohibited concomitant medications) are provided?*Contraindications to G-CSF treatment (WBC> 10000/μl (normal range: 4000-10000/μl be), a hematocrit> 48% or platelet count <150000/μl (normal range: 150000-450000/μl) at initial blood tests; known allergy to G-CSF, a congenital neutropenia, an alcohol dependence, pregnancy); severe and untreated medical conditions (severe rheumatoid arthritis, severe liver and kidney disease, recurrent venous thrombosis, recurrent pulmonary embolism, a refractory arterial hypertension, severe blood disease and myocardial infarction); Mini-Mental State Examination > 26.

*- The risk-benefit ratio: What benefits are expected from the results of the study*

 *a) for the participants?* The trial participants may benefit from a possible improvement of the cognitive and motor deficits.

 *b) for medicine?* The long-term benefit is to improve the pharmacological treatment of stroke sequalae, which have limited therapeutic options.

c) *for science?* The direct benefit for science is a better understanding of the function of neurogenesis and neuroprotection in the adult human brain and its pharmacological interference. The results could likely transferred to other diseases with cognitive and motor deficits.

*- Risks of the study*: *What kind of risks?*Common side effects (> 10%) of G-CSF are headache, musculoskeletal pain, fatigue, leukocytosis and thrombocytopenia. Both head and bone pain can be successfully treated with paracetamol. Less frequent (<10%) side effects that might occur are elevated alkaline phosphatase and increased lactate dehydrogenase. Rare (<1%) side effects are allergic reactions, a ruptured spleen, increased SGOT, hyperuricaemia or worsening of rheumatoid arthritis. The side effects are related to the administered dose and the time of application.

*- Have criteria been defined which lead to an interruption of the whole trial? If so, what criteria?*

The study will be terminated as soon as indicated by the occurrence of serious adverse events described above. These include in particular a splenic rupture, thromboembolic events and severe allergic reactions.
 *- How are the trial participants be informed about the nature, significance and scope of the study?*
The trial participants are informed about the nature, significance and scope of the study in person and on the basis of standardized consent forms by the investigator.

*- How will the trial participants declare their consent to participate in the study?*The consent will be carried out by signature on the patient's consent form.

*- What kind of drugs are tested? (Please include the following: name, description, chemistry of the substance, pharmacology, toxicology, pharmacokinetics)*The Granulocyte - Colony Stimulating Factor (G-CSF) is tested. It is a naturally occurring glycoprotein that is produced artificially by recombinant DNA technology. There is a glycosylated (Lenograstim) and a non-glycosylated (filgrastim) form. Lenograstim is produced in ovary cells from Chinese hamsters and Filagrastim in the bacterium Escherichia coli. In our study Filgrastim (r-= metHuG-CSF) is used. The clearance of filgrastim follows a first-order kinetics. The half-life in serum is about 3-4h with a clearance of about 0.6 ml per minute and kg. The maximum serum concentration after subcutaneous administration is achieved after 2-8h. In healthy blood donors a dose of 10 or 2x5 g / kg KG / day is given to mobilize peripheral blood stem cells from bone marrow. The “peak number" of the CD34 + - cells can be detected 5 days after leukapheresis.

A saline solution will be used as placebo substance.

- *Is the used substance approved by the Federal Institute for Drugs and Medical Devices?*
The test substance is already used as a drug for different indications. It is also used in healthy people.

**References**

Aberg M, Aberg D, Hedbäcker H, Oscarsson J, Eriksson P. Peripheral Infusion of IGF-I Selectively Induces Neurogenesis in the Adult Rat Hippocampus. J. Neurosci. 2000, 20(8): 2896-2903.

Chung YH, Kim SI, Joo KM, Kim YS, Lee WB, Yun KW, Cha CI. Age-related changes in erythropoetin immunoreactivity in the cerebral cortex and hippocampus of rats. Brain Research 2004, 1018. 141-146.

Dame C, Juul SE, Christensen RD. The Biology of Erythropoetin in the Central Nervous System and Its Neurotrophic and Neuroprotective Potential. Biol Neonate 2001, 79: 228-235.

Döbrössy MD, Drapeau E, Aurousseau C, Moal ML, Piazza PV, Abrous DN. Differential effects of learning on neurogenesis: learning increases or decreases the number of newly born cells depending on their birth date. Molecular Psychiatry 2003, 8: 974-982.

Drapeau E, Mayo W, Aurousseau C, Moal ML, Piazza P-V, Abrous DN. Spatial memory performances of aged rats in the water maze predict levels of hippocampal neurogenesis. PNAS 2003, 100 (24): 14385-14390.

Ghezzi P, Brines M. Erythropoetin as an antiapoptotic, tissue-protective cytokine. Cell Death and Differentiation 2004, 11: 37-44.

Gibson CL, Bath PM, Murphy SP. G-CSF reduces infarct volume and improves functional outcome after transient focal cerebral ischemia in mice. J Cereb Blood Flow Metab. 2005, 19: [Epub ahead of print]

Hackel ME, Wolfe GA, Bang SM, Canfield JS. Changes in hand function in the aging adult as determined by the Jebsen Test of Hand Function. Phys Ther. 1992, 72(5): 373-7.

Jebsen RH, Taylor N, Trieschmann RB, Trotter MJ, Howard LA. An objective and standardized test of hand function. Arch Phys Med Rehabil 50(6): 311-9

Jelkmann W, Wagner K. Benefical and ominous aspects of the pleotropic action of erythropoetin. Annals of Hematology 2004

Jin K, Sun Y, Xie L, Peel A, Mao XO, Batteur S, Greenberg DA. Directed migration of neuronal precursors into the ischemic cerebral cortex and striatum. Molecular and Cellular Neuroscience 2003, 24: 171-189.

Kempermann G, Gage FH. Closer to neurogenesis in adult humans. Nature Medicine 1998, 4(5): 555-557.

Kempermann G, Wiskott L, Gage FH. Functional significance of adult neurogenesis. Current Opinion in Neurobiology 2004, 14: 186-191.

Lezak MD Neuropsychological assessment. New York, Oxford: Oxford University Press 2004.

Maiese K, Li F, Zhong Chong Z. New Avenues of Exploration for Erythropoetin. JAMA 2005, 293: 90-95.

Nilsson M, Perfilieva E, Johansson U, Orwar O, Eriksson PS. Enriched Environment Increases Neurogenesis in the Adult Rat Dentate Gyrus and Improves Spatial Memory. J Neurobiol. 1999, 39(4): 569-578.

Nunes MC, Roy NS, Keyoung HM, Goodman RR, McKhann G, Jiang L, Nedergaard M, Goldmann SA. Identification and isolation of multipotential neural progenitor cells from the subcortical white matter of the adult human brain. Nature Medicine 2003, 9(4): 439-447.

Roy NS, Wang S, Jiang L, Benraiss A, Harrison-Restelli C, Fraser R, Couldwell WT, Kawaguchi A, Okano H, Nedergaard M, Goldmann SA. In vitro neurogenesis by progenitor cells isolated from the adult human hippocampus. Nature Medicine 2000, 6(3). 271-277.

Schäbitz WR, Kollmar R, Schwaninger M, Juettler E, Bardutzky J, Schölzke MN, Sommer C, Schwab S. Neuroprotective Effect of Granulocyte Colony-Stimulating Factor After Focal Cerebral Ischemia. Stroke 2003, 34: 745-751.

Shyu W-C, Lin S-Z, Yang H-I, Tzeng Y-S, Pang C-H, Yen P-S, Li H. Functional Recovery of Stroke Rats Induced by Granulocyte Colony-Stimulating Factor – Stimulated Stem Cells. Circulation 2004, 110: 1847-1854.

Sirén AL, Ehrenreich H. Erythropoetin – a novel concept for neuroprotection. Eur Arch Psychiatry Clin Neurosci 2001, 251: 179-184.

Strong M, Rosenfeld J. Amyotrophic lateral sclerosis: a review of current concepts. Amyotroph Lateral Scler Other Motor Neuron Disord. 2003, 4(3): 136-143.

Van Praag H, Christie BR, Sejnowski TJ, Gage FH. Running enhances neurogenesis, learning and long-term potentation in mice. Neurobiology 1999, 96(23): 13427-13431.

Van Praag H, Schinder AF, Christie BR, Toni N, Palmer TD, Gage FH. Functional neurogenesis in the adult hippocampus. Nature 2002, Vol 415: 1030-1034.

Wagner JP, Black IB, Bloom ED. Stimulation of Neonatal and Adult Brain Neurogenesis by Subcutaneous Injection of Basic Fibroblast Growth Factor. J. Neurosci. 1999, 19(14): 6006-6016.

Wang L, Zhang Z, Wang Y, Zhang R, Chopp M. Treatment of Stroke With Erythropoetin Enhances Neurogenesis and Angiogenesis and Improves Neurological Function in Rats. Stroke 2004, 35: 1732-1737.

Zhang J, Li Y, Cui Y, Chen J, Lu M, Elias SB, Chopp M. Erythropoetin treatment improves neurological functional recovery in EAE mice. Brain Research 2005, 1034: 34-39.
